# Reduced Graphene Oxides: Influence of the Reduction Method on the Electrocatalytic Effect towards Nucleic Acid Oxidation

**DOI:** 10.3390/nano7070168

**Published:** 2017-07-04

**Authors:** Daniela F. Báez, Helena Pardo, Ignacio Laborda, José F. Marco, Claudia Yáñez, Soledad Bollo

**Affiliations:** 1 Centro de Investigación de Procesos Redox, CiPRex, Facultad de Ciencias Químicas y Farmacéuticas, Universidad de Chile, Sergio Livingstone 1007, Independencia, Santiago 8380492, Chile; d.baez@ciq.uchile.cl (D.F.B.); cyanez@ciq.uchile.cl (C.Y.); 2 Advanced Center for Chronic D (ACCDiS), Facultad de Ciencias Químicas y Farmacéuticas, Universidad de Chile, Sergio Livingstone 1007, Independencia, Santiago 8380492, Chile; 3 Facultad de Química, Universidad de la República de Uruguay, Avenida General Flores 2124, Montevideo 11800, Uruguay; hpardo@fq.edu.uy (H.P.); ilaborda@fq.edu.uy (I.L.); 4 Instituto de Química Física Rocasolano, CSIC, calle Serrano 119, 28006 Madrid, Spain; jfmarco@iqfr.csic.es

**Keywords:** graphene, reduced graphene oxide, glassy carbon electrode, SECM, DNA oxidation

## Abstract

For the first time a critical analysis of the influence that four different graphene oxide reduction methods have on the electrochemical properties of the resulting reduced graphene oxides (RGOs) is reported. Starting from the same graphene oxide, chemical (CRGO), hydrothermal (hTRGO), electrochemical (ERGO), and thermal (TRGO) reduced graphene oxide were produced. The materials were fully characterized and the topography and electroactivity of the resulting glassy carbon modified electrodes were also evaluated. An oligonucleotide molecule was used as a model of DNA electrochemical biosensing. The results allow for the conclusion that TRGO produced the RGOs with the best electrochemical performance for oligonucleotide electroanalysis. A clear shift in the guanine oxidation peak potential to lower values (~0.100 V) and an almost two-fold increase in the current intensity were observed compared with the other RGOs. The electrocatalytic effect has a multifactorial explanation because the TRGO was the material that presented a higher polydispersity and lower sheet size, thus exposing a larger quantity of defects to the electrode surface, which produces larger physical and electrochemical areas.

## 1. Introduction

The study of graphene has become the subject of intense research over the last decade due to its unusual physical, chemical, and mechanical properties, and its potential applications in different fields. It has a very high chemical stability and aspect ratio and its specific surface area is approximately 2675 m^2^/g (almost twice that of carbon nanotubes) [1]. Graphene electrodes present several advantages, such as wide potential windows, inertness as a catalyst, and good electro-catalytic activity. However, it presents a limited number of electroactive sites on the surface, which limits the sensitivity of a potential biosensor.

Graphene oxide (GO), a graphene derivative material is a graphene sheet functionalized from both sides with hydroxyl, epoxide, and carbonyl groups and the edges are functionalized with carboxyl groups, which gives this material a strong hydrophilic character. GO has also been widely reported as a biosensor material due to its high chemical and electrochemical activity. However, these oxidized groups break the conjugated network and π-electron conductivity [2].

Among the graphene derivatives, reduced graphene oxide (RGO) is believed to be one of the best candidates due to its reasonably reduced number of functionalities, a large number of remaining electroactive sites, and the structural similarity with graphene. RGO also offers a wide range of advantages, such as cheap and reliable preparation, good reaction yields, large surface areas, the possibility of functionalization, and good biocompatibility [3].

Different methods of reducing the GO precursor material to produce RGO have been published [4,5]. The final goal in all cases is to restructure the characteristic graphitic sp^2^ network. However, the reduction methods lead to different properties of the resulting RGOs, which clearly can influence the electrochemical response of the RGO-modified electrodes [4,6]. The reduction of GO results in materials with typical C/O relations of 12/1; to do this reduction, different alternatives can be applied that would result in different kinds of graphene materials due to the presence of residual functional groups and different types of defects in variable proportions. The presence of residual functional groups and defects must be considered because they will have consequences on the existence of structural, chemical, and physical differences with respect to pure graphene and will determine the physicochemical behaviours of the final product. Among the properties that are modified are the conductivity, chemical reactivity, mechanical properties, surface area, and electrocatalytic performance, the last of which is particularly important for this work [7,8,9].

Among the first reported methods to reduce GO was the chemical procedure using sodium borohydride (NaBH_4_) or hydrazine (N_2_H_2_). In both cases, the reduction is not complete; i.e., oxygenated functionalities remain in the structure and introduce impurities from the reducing agents [10]. Shin et al. realized the comparison between the reducing effect of N_2_H_4_ and NaBH_4_ on GO films and found that the resistance of NaBH_4_-reduced films was much lower than that of N_2_H_4_-reduced films, even though both films showed a similar proportion and composition of oxygenated species. They attributed this behaviour to the formation of nitrogen groups from the hydrazine in the graphene sheets [11].

Pumera et al. reported the comparative use of different chemically modified graphenes on electrochemical devices in several papers [12,13]. In 2014, they reported on the surface composition of chemically reduced graphene oxides resulting from the use of different masses of sodium borohydride (NaBH_4_) and the effect that this surface composition has on the electron transfer properties [14]. They concluded that graphene materials with a greater degree of reduction exhibit improved conductivities. Nevertheless, when the chemical RGOs (CRGOs) were tested against different redox molecules, they found that the dependency of the heterogeneous electron transfer on the oxygen content in CRGO is complex and that multiple concurrent effects occur.

In contrast, thermal annealing is the most energetic and rapid method to obtain RGOs, with temperatures of approximately 1000 °C that produce pressures of ~130 MPa [15]. This exothermic method has better results than the chemical method because it removes a major part of the oxygen functionalities. However, it inevitably leaves defects, such as carbon sp^3^ and vacancies, in its structure [16]. Recent work was devoted to comparing the effect of the thermal reduction temperature to the produced TRGO [17]. The authors demonstrate that there is a structural disorder and that the liberation of the pores caused by the removal of intercalated water and labile oxygen functional groups was favoured at temperatures of ~300 °C. However, when higher temperatures were used (900 °C), new defects on the RGO surface appeared and provided additional access to the internal space between the folds and sheets of the TRGO structure. 

Sakinyte et al. prepared three kinds of thermally produced RGOs (TRGOs) using a vertical thermal reduction system with the goal of creating reagentless amperometric d-fructose biosensors. They reported high sensitivities of the same order as other d-fructose sensors. However, one of the RGOs, TRGO1, exhibited the highest sensitivity, and the authors concluded that the large quantity of functional groups present in the TRGO1 fraction were able to immobilize the enzyme properly and allowed the electron transfer reactions to occur. Additionally, they concluded that the surface area of the modified electrode does not play a crucial role in the effectiveness of the electron transfer [18].

As an alternative to these rough and defective methods, the hydrothermal and electrochemical reduction methods [5,19] appear as green methods because they use moderate temperatures and pressures in aqueous solutions [20]. The effects that the morphology and defect density have on the electron transfer of electrochemically reduced graphene oxide were published and indicated that both defect densities and edge planes of ERGO play crucial roles in the electron transfer kinetics [21].

In contrast, the electrochemical detection of nucleic acid sequences uses a single stranded sequence, called oligonucleotide (Oligo) as a probe to detect a specific and complementary target sequence (hybridization event) [22]. This alternative is one of the most interesting and novel techniques tested because it is simple and inexpensive compared to the usual analyses in molecular biology [23]. Attachment strategies using covalent or sorption attachment and the class of graphene derivatives used have also been studied on these planar structures [24]. In addition, several studies about DNA biosensors have been reported for GO [25,26,27] and RGO [28], but no comparison between the GO/RGO materials was performed. In a previous work, we demonstrated that CRGOs show clear electrochemical improvement compared to GO when used against dsDNA oxidation [29].

To our knowledge, although several studies have used RGO, no comparative studies of the influence of different graphene oxide reduction methods on the electrochemical behaviour of DNA oxidation have been conducted and, more importantly, using the same GO as the starting material. 

In this work, the Hummers modified method is used to obtain GO, in which the oxidative exfoliation of graphite is achieved by the use of concentrated sulfuric acid and potassium permanganate under controlled reaction conditions. In this method, the active species are dimanganese heptoxide (Mn_2_O_7_) and permanganyl cation ($MnO_{3}^{+}$), which are formed from the reaction of KMnO_4_ with concentrated H_2_SO_4_ [30]. We then complete a comparative study on the effect of four different GO reduction methods, i.e., thermal, electrochemical, chemical, and hydrothermal, on the electrooxidation of an oligonucleotide using a non-covalent immobilization process. Reduced graphene oxide derivatives were characterized by Transmission Electron Microscopy (TEM), Scanning Electron and Electrochemical Microscopy (SEM, SECM), Brunauer-Emmett-Teller (BET) analysis, X-ray powder Diffraction (XRD), X-ray Photoelectron (XPS), and Raman spectroscopy. Finally, using differential pulse voltammetry, the electrooxidation of the oligonucleotide was evaluated. The final goal was to determine which of the RGOs present the best characteristics to be used in the future development of DNA hybridization biosensing applications. 

## 2. Experiment

All chemical reagents used in this work were of analytical grade. The graphite powder used as the starting graphite material was from Fluka (Ronkonkoma, NY, USA). All solutions prepared for synthesis and electrochemical measurements were made using Milli-Q water (Millipore Co., Bedford, MA, USA) and were carried out at room temperature. A Pt wire and Ag/AgCl electrode were used as auxiliary and reference electrodes, respectively, in all electrochemical experiments. All potentials refer to this reference electrode.

### 2.1. Synthesis of Graphene Oxide Derivatives

**Graphene oxide (GO).** GO was prepared according to the modified Hummers and Offeman’s method [9]. First, 1.0 g of graphite powder in 50 mL of concentrated H_2_SO_4_ was placed in an ice bath under continuous stirring and stored for 30 min. Then, 4.0 g of KMnO_4_ and 150 mL of distilled water were slowly added, and the solution was stirred for one hour. Subsequently, 5.0 mL of H_2_O_2_ was added to reduce the residual permanganate to bring the colour of the mixture from black to brown. The suspension was left overnight to decant with 100 mL of HCl (5% *v*/*v*). The resulting suspension was centrifuged and washed several times with HCl (5% *v*/*v*) and water until a neutral pH was achieved and the colour of the mixture turned to dark brown. The product was dried in a stove at 50 °C for one day.

The oxidation reaction was monitored by XRD to observe the complete pattern disappearance of the reflection (002) at 2θ = 26.50°, which is characteristic of graphite and the appearance of a new peak at 2θ = 10.2°.

**Chemically Reduced GO (CRGO).** First, 200 mg of GO was dispersed in 200 mL of distilled water, and the pH was adjusted to 9 with sodium carbonate; then, the dispersion was sonicated for 30 min using an ultrasonic tip. The chemical reaction was carried out in an oil bath at 90 °C under continuous stirring while 1.6 g of NaBH_4_ was added to the mixture. The reaction was complete after 72 h. Finally, the product was washed by successive centrifugations with Milli-Q water, until obtaining a neutral pH, and was dried in a stove at 50 °C [5]. 

**Thermally Reduced GO (TRGO).** A sample of solid and dried GO was subjected to thermal shock. The GO was placed in a porcelain boat, introduced in a furnace and heated up to 950 °C under an inert flux of nitrogen to avoid burning the sample [5]. Then, 30 s was selected as the optimal time because the GO is permitted to reduce completely without consuming the sample. 

**Hydrothermally Reduced GO (hTRGO).** First, 150 mL of an aqueous solution at pH 9 containing 75 mg of GO was introduced into an autoclave system consisting of a Teflon recipient inside of a sealed stainless steel camera and was maintained overnight at 160 °C. The resulting hTRGO appeared at the bottom of the autoclave in the form of a black solid that was finally oven dried at 50 °C [5].

**Electrochemically Reduced GO (ERGO).** First, 4.0 mg of GO was dispersed in 1.0 mL of distilled water and sonicated for over 30 min. Then, 40 µL of this GO dispersion was deposited onto a glassy carbon disc previously polished with 0.3 and 0.05 µm alumina for 20 s and then rinsed with water, and it was left to dry in an oven at 50 °C. This material was used as the working electrode. The electrochemical reduction was carried out using a CHI 660 potentiostat (CH Instrument Inc., Austin, TX, USA) with a conventional 3-electrode system. One hundred cyclic voltammograms in 0.5 M NaCl using a potential range between 0 and −1.5 V (vs. Ag/AgCl reference electrode) at a scan rate of 0.1 V·s^−1^ were performed. All cycles were performed under nitrogen atmosphere in a one-cell compartment [31]. 

### 2.2. Characterization of RGOs

**X-ray photoelectron spectroscopy (XPS)**. XPS spectra were recorded with a CLAM-2 electron analyzer and non-monochromatic Mg Kα radiation under a base pressure greater than 5 × 10^−9^ torr. The spectra were acquired using a constant pass energy of 20 eV, and the binding energies were accurate to within ±0.2 eV. The relative atomic concentrations were obtained from the integration of the peak area after Shirley background subtraction using tabulated atomic sensitivity factors.

**Scanning electron microscopy (SEM)**. SEM images were obtained with an F50 instrument. For the measurements, samples were deposited in solid form on a glassy carbon disc (12.7 mm).

**Raman spectroscopy**. Raman spectra were registered with a Renishaw micro-Raman system (RM1000) equipped with a 785 nm excitation laser line. The spectra were scanned in the 1800–200 cm^−1^ region. 

Specific surface areas were obtained by the Brunauer-Emmett-Teller (BET) method. BET areas were determined using the Micromeritics ASAP 2010 Surface Area and Porosity Analyser (Micromeritics Instrument, Norcross, GA, USA). The samples were pre-treated at 200 °C for 2 h and analysed by nitrogen adsorption measurements.

### 2.3. Electrochemical Experiments

#### 2.3.1. Preparation of the Modified Electrode (GCE/RGO)

Prior to the electrochemical measurements, the GCE (3 mm diameter) was cleaned by polishing with 0.3 and 0.05 µm alumina slurries for 20 s. Next, 1 mg of each RGO was dispersed in a phosphate buffer solution (PBS) of 0.1 M at a pH 7.0 or a 1.0% *w*/*v* chitosan solution prepared in 1.0% *v*/*v* acetic acid solution by sonication for 15 min for the subsequent experiments of cyclic voltammetry (CV) and differential pulse voltammetry (DPV), respectively. The final concentration was 1 mg/mL, and the immobilization of RGOs was performed by casting the GCE with 10 µL of each RGO dispersion and drying the modified electrode for 15 min in a stove at 50 °C. 

#### 2.3.2. Cyclic Voltammetry and Differential Pulse Voltammetry

The experiments were performed using a CHI 660 potentiostat and conventional 3-electrode system. The experiments were carried out in PBS (0.10 M, pH 7.0) or ferricyanide solution (2 mM with KCl 0.1 M) at room temperature under nitrogen atmosphere. RGO modified glassy carbon electrodes were obtained as previously described. DPV conditions: pulse amplitude 0.05 V, pulse width 0.05 s, pulse period 0.2 s, potential increment 0.004 V.

#### 2.3.3. Scanning Electrochemical Microscopy (SECM)

The SECM experiments were performed using a CHI 900 bipotentiostat (CH Instruments Inc., Austin, TX, USA). A 10-µm diameter carbon electrode (homemade) was used as the SECM tip and was polished as previously described. An RGO modified glassy carbon electrode was used as a substrate electrode. The experiments to obtain the SECM images were carried out in 0.10 M PBS with a pH of 7.0, using ferrocene methanol (FcOH) as the redox mediator. The tip and substrate potentials were held at 0.500 V and 0.000 V, respectively. Thus, at the tip, FcOH is oxidized and forms Fc^+^OH, and at the substrate, the reduction of the Fc^+^OH occurs, regenerating the parent FcOH to allow the feedback between the electrodes.

A series of 100 µm × 100 µm, constant-height SECM images were recorded at a tip scan rate of 1 µm/s. The results are presented as the normalized current (*I*_T_) by standardizing the experimental feedback current (*i*_T_) using the steady-state current obtained when the tip was far from the substrate (*i*_T_ = 4naFDC), i.e., *I*_T_ = *i*_T_/*i*_T_ [32].

### 2.4. Electrooxidation of Nucleic Acid

The modified electrode (GCE/RGO) was immersed in a 3.0% *v*/*v* GTA solution for 2 s [33]; then, the electrode was washed by dipping it in a 0.2 M formate buffer solution (FBS) at pH 5.0 for 20 s. The given electrode was immersed in stirred FBS containing a 15 µM Oligo solution (5′ATT GCT CGA CGT ACG CAG TTA 3′) to adsorb the oligonucleotide via accumulation at the open circuit potential for different durations. The electrode containing the adsorbed Oligo was washed for 10 s with the PBS, and a DPV was performed in PBS at pH 7. The anodic current observed corresponded to the guanine oxidation, which was used as the analytical signal.

## 3. Results and Discussion

### 3.1. Evaluation of the Efficiency of Reduction Methods

X-ray Photoelectron spectroscopy (XPS) was the technique selected to perform the chemical characterization of both the starting GO and the reduced materials. Figure S1 in the Supplementary Materials shows the wide scan spectra recorded from graphite, GO, and the other reduced samples. They are dominated by intense carbon and oxygen signals (except for the case of graphite, in which the oxygen signal is really small, likely due to a small adventitious contamination contribution). Inspection of this figure shows, as expected, that the intensity of the oxygen peak is smaller in the RGOs than in the GO and that this intensity varies depending on the reduction method used. Therefore, the O/C atomic ratio can be taken as an indication of the extent of reduction achieved by each synthesis method. Thus, the O/C atomic ratio varies from 0.45 for GO to 0.28 for CRGO, 0.19 for hTRGO, 0.17 for TRGO, and 0.15 for ERGO. Consequently, the results indicate that CRGO is a less efficient method for reduction and that the other three achieve similar degrees of reduction.

**Figure 1:**
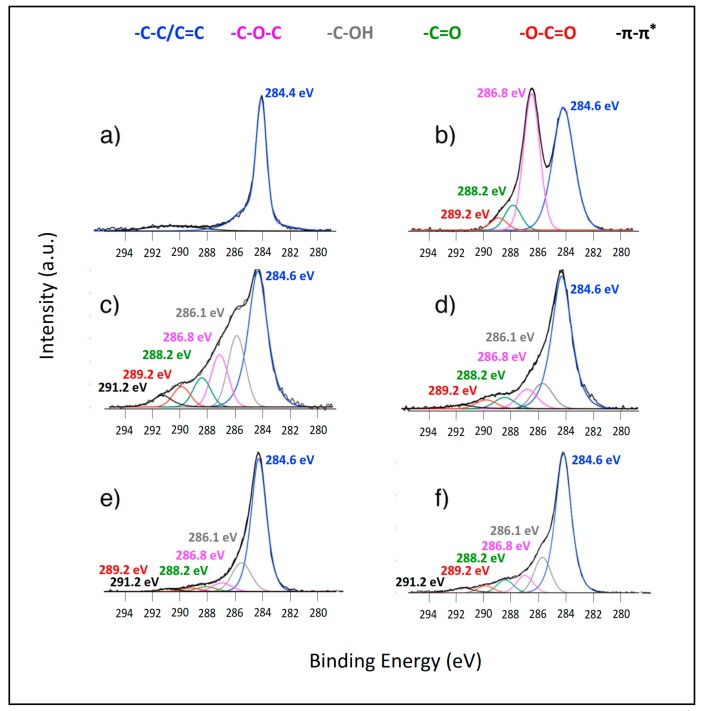
X-ray photoelectron spectroscopy (XPS), C 1s spectra of (**a**) graphite, (**b**) GO; (**c**) CRGO; (**d**) hTRGO; (**e**) ERGO and (**f**) TRGO.

A careful analysis of the high-resolution C 1s spectra provides information about the surface carbon functional groups present in each of the materials examined (Figure 1). In the case of pristine graphite, the C 1s spectrum contains a very intense, asymmetric, narrow peak at 284.4 eV and a broad satellite contribution at higher binding energies, which is characteristic of carbon with sp^2^ hybridization, as expected for the graphitic structure (Figure 1a). The spectrum recorded from GO matches that reported in the literature [32] and indicates that the oxidation method employed was successful. The spectrum was fitted considering the same model used in reference [34], considering four different contributions. The contribution located at 284.6 eV can be associated with the C–C/C=C groups, whereas those observed at 286.8 eV, 288.2 eV, and 289.2 eV correspond to the C–O, C=O, and O–C=O groups, respectively. The spectra of the reduced materials were all different, and more contributions were needed to adequately fit the experimental data. Therefore, in addition to the four contributions mentioned above, it was necessary to include one at 286.1 eV, which can be associated with the C–OH groups, and a broad π–π* satellite at approximately 291.2 eV [35,36]. The intensities of these oxygen-containing groups varied considerably with respect to those shown in the spectrum of GO and from spectrum to spectrum. The combined contribution of the oxygen-containing groups could be taken as a measure of the extent of reduction achieved; i.e., the larger the percentage of oxygen-containing species is, the smaller the extent of reduction achieved. Within the error of the quantitative experimental determination, the trend observed in Figure 1 correlates well with the O/C ratio obtained from the wide scan spectra. 

In summary, the XPS data show that the four reduction methods produce decreases in the oxygen content, but that the chemical procedure is the least efficient at reducing GO, whereas the other three offer more efficient and similar results.

### 3.2. Electrochemical Characterization of RGOs Modified Electrodes

**Figure 2:**
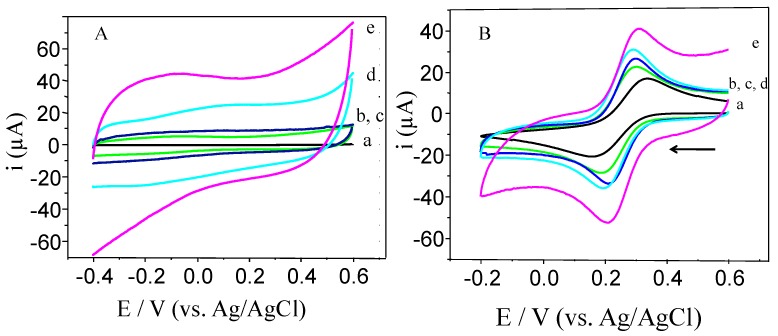
Cyclic voltammograms obtained in (**A**) 0.10 M PBS, pH 7.0 and (**B**) 2 mM ferricyanide solution, KCl 0.1 M. GCE (a, black line), GCE/CRGO (b, green); GCE/hTRGO (c, blue); GCE/ERGO (d, cyan), and GCE/TRGO (e, magenta) (1.0 mg/mL) dispersed in PBS at pH 7.0. Scan rate, 0.1 V/s.

Figure 2 shows cyclic voltammograms of the GCE/RGOs modified electrodes compared to the bare glassy carbon electrode in a phosphate buffer solution (PBS) and a ferricyanide solution. In PBS (Figure 2A), the bare GCE (curve a) shows a very small capacitive current, and no faradic current is observed in the applied potential range. In contrast, when the surface is modified with RGOs (curves b–e), the capacitive currents increase as a result of the presence of the RGO sheets. However, great differences are clearly observed among the different RGOs. The values obtained at 0.30 V are 40.0, 22.0, 8.70, and 4.80 µA for TRGO, ERGO, hTRGO, and CRGO, respectively. The capacitive current values give insight into the electrochemical area of the resulting electrodes. The CV curves show a typical electric double layer behaviour without any redox processes associated, except in the case of TRGO (curve e), where a remaining oxygen signal can be appreciated at −0.40 V, and in the case of ERGO (curve b), where a small electrochemical signal is observed at 0.100 V, indicating that the small amount of oxygenated functional groups observed with XPS are electrochemically active [37].

Using Fe(CN)_6_^3−/4−^, a redox probe sensitive to the surface chemistry of carbon-based electrodes via an out-sphere electron transfer process [38], increases in the peak current responses and decreases in the peak-to-peak potential separations (ΔE_P_) are observed at the GC/RGO electrodes (Figure 2B). The larger current response of Fe(CN)_6_^3−/4−^ indicates an increase in the number of electrochemically active sites, and a lower ΔE_P_ indicates faster electron transfer kinetics, and TRGO exhibited the better electrochemical performance. 

**Figure 3:**
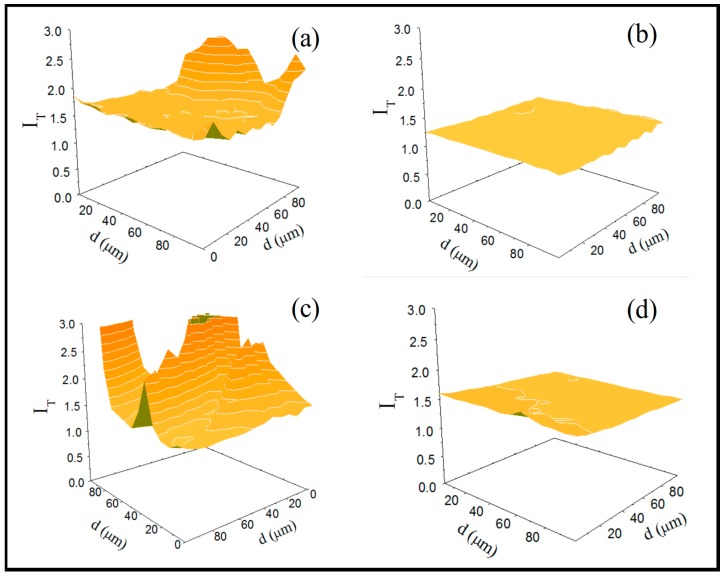
Scanning Electrochemical Microscopy, SECM surface-plot images of (**a**) GCE/CRGO; (**b**) GCE/hTRGO; (**c**) GCE/ERGO; and (**d**) GCE/TRGO. Experimental conditions: 5.0 × 10^−4^ M FcOH, supporting electrolyte: 0.10 M phosphate buffer solution with a pH of 7.00, ET = 0.050 V, ES = 0.000 V, UME scan rate: 10 µm·s^−1^.

The GCE modified with the RGO derivatives were also evaluated by SECM using FcOH as a redox probe, which permits us to obtain information about the distribution of the nanomaterial on the GCE surface, i.e., the topography, as well as the localized electroactivity of the resulting electrode. Figure 3 displays one representative 3D surface image from at least six for the GCE/CRGO, GCE/hTRGO, GCE/ERGO, and GCE/TRGO electrodes. Because an unmodified GCE has a standard normalized current (*I*_T_) of 1.25 [32], two patterns are clearly observed when the RGOs were immobilized onto the CGE. First, the GCE/CRGO and GCE/ERGO surfaces (Figure 3a,b) show heterogeneous behaviours, thus revealing the existence of non-uniform distributions or agglomerates of RGOs on the surface. In contrast, GCE/hTRGO and GCE/TRGO surfaces (Figure 3c,d) show completely homogenous topographies, suggesting that the RGOs were efficiently dispersed and distributed on the GCE surfaces. Furthermore, for the electroactivity of the resulting electrodes, the GCE/hTRGO shows a normalized current similar to that informed for bare GCE, and the GCE/TRGO modified electrode presents a higher value, with a normalized current close to 1.5, indicating that the TRGO is the reduced graphene oxide derivative that produces the most homogeneous and electroactive surface.

In conclusion, the electrochemical characterization of the GCE electrodes modified with different RGOs reveals completely different properties. First, the chemical and electrochemical RGOs present poor dispersion properties that generate non-homogeneous surfaces and low capacitive currents that reflect smaller electroactive areas. However, the hydrothermal and thermal RGOs generate homogeneous electroactive surfaces, but the thermal RGO presents higher capacitive currents and therefore a higher electroactive area.

### 3.3. Structural and Physical Characterisation of RGOs

**Figure 4:**
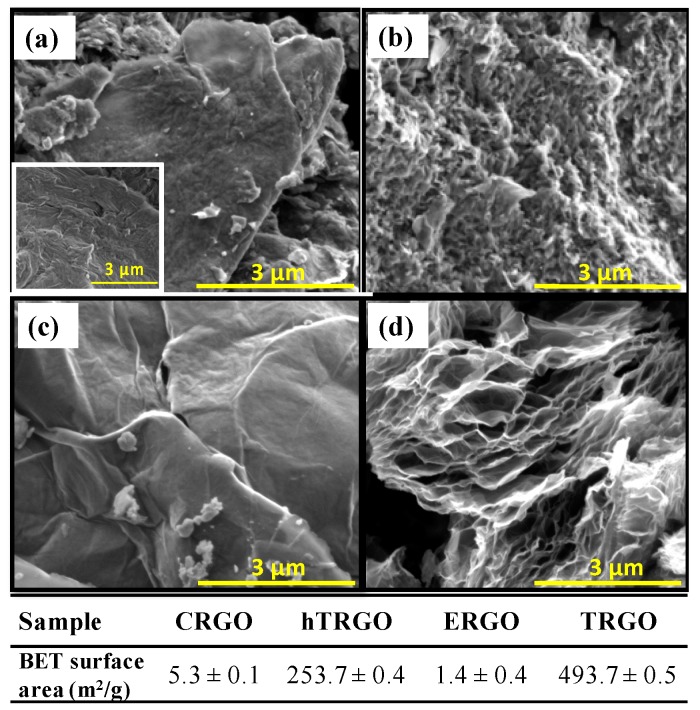
SEM images obtained for (**a**) CRGO; (**b**) hTRGO; (**c**) ERGO; and (**d**) TRGO. Scale bar: 3 µm. Inset image (**a**) SEM of the starting GO. Table below is the data of specific surface area.

The reduction methods produce a clear effect on the morphology of the resulting RGOs. Scanning Electron Microscopy (SEM) images of CRGO, hTRGO, ERGO, and TRGO are shown in Figure 4a–d. CRGO shows a compact and corrugated surface with particles smaller than those observed for GO (inset Figure 4a). hTRGO presents a homogeneous porous morphology, while in the case of ERGO, a film morphology is observed. TRGO shows a porous sponge morphology with clearly visible separated sheets. Figure 4 reveals that the hydrothermal and thermal reduction processes are accompanied by a pronounced decrease in the structure compactness. 

The specific surface areas (SSAs) obtained for each RGO are also given as an insert in Figure 4. Data were modelled using the multilayer adsorption model proposed by Brauner, Emmet, and Teller (BET) and are consistent with the SEM results. TRGO presents the highest surface area (493.7 ± 0.5 m^2^/g) followed by hTRGO (253.7 ± 0.4 m^2^/g). The areas for the other two RGOs, which correspond to the chemically and electrochemically reduced materials, are two orders of magnitude smaller, with values close to those reported for graphite [39,40,41]. Despite the high values of TRGO, the value is significantly lower than the theoretical surface area (2630 m^2^/g) reported for an individual graphene sheet [42], indicating that the RGO samples are composed of several layers of graphene sheets.

Transmission electron microscopy (TEM) images allows the effect that the reduction methods have on the size and morphology of the sheets to be verified. Figure S2 (Supplementary Materials) shows the images of GO with a laminar structure and a sheet size larger than 20 µm; the high contrast background indicates a significant overlap of the sheets. For CRGO and hTRGO, flat sheets were observed with sizes greater than 10 µm and a low contrast, indicating low overlapping. Finally, for TRGO and ERGO, corrugated surfaces were observed with sizes lower than 1 µm. This response has been associated in the literature with defects or vacancies present in the planar base of the structure, which results in a break or curvature of the sheets [43,44].

These results correlate well with the electrochemical characterization because more compact RGOs demonstrate poor dispersability that produce agglomeration and non-homogeneous electrodic surfaces. In contrast, TRGO presents a more porous material and lower sheet size, which are reflected in an electroactive and homogeneous electrochemical surface.

X-ray powder diffraction (XRD) patterns of the four reduced graphene oxides are presented in Figure S3. For comparative purposes, the patterns of pristine graphite and graphene oxide are included. The XRD pattern of graphite (Figure S3a) shows a sharp main diffraction peak at 2θ = 26.50° indicative of an interlayer distance of 0.34 nm. When graphite is oxidized, its characteristic diffraction peak starts decreasing in intensity until it completely disappears. Simultaneously, a new peak at 2θ = 10.2° (Figure S3b) starts appearing. The new peak, which is not as sharp but is intense, is equivalent to an interlayer distance of 0.86 nm. The increase in the interlayer distance is due to the incorporation of oxygenated functionalities during the oxidation process and to the intercalation of water molecules between the new hydrophilic GO sheets. The complete disappearance of the peak at 2θ around 26° indicates that no residual graphitic structure remains in GO [45]. After the reduction processes, the diffractograms show broad diffraction peaks at 2θ around 24°, close to that shown by graphite, coinciding with the disappearance of the diffraction peak of GO. This behaviour is present in all of the reduced samples (Figure S3c–f). The presence of the peak at 2θ around 24° confirms that all reduction methods induce the restoration of the graphitic structure with a corresponding *d* spacing of 0.35 nm for CRGO, 0.37 nm for hTRGO, 0.36 nm for TRGO, and 0.37 for ERGO, which is characteristic of the synthesised RGOs [16]. However, the broad peaks indicate that the new RGOs show a poorer crystalline character than graphite. The presence of a couple of sharp peaks in the XRD pattern of ERGO at 2θ = 31.7° and 42.5° corresponds to the presence of NaCl used as the supporting electrolyte in the electrochemical cell for the synthesis of this particular material. 

Several authors report the same behaviour in their synthesis processes but with differences in the complete disappearance of the signal corresponding to GO after the reduction process [46]. Bosch et al. reduced GO under hydrothermal conditions using different pH values, and the resulting RGOs present clear residual peaks at 2θ around 12° [19]; using a hydrothermal reduction method but at different temperatures, they also obtained XRD patterns without the complete disappearance of the GO peak. In our case, the four reduction methods produce RGOs without the presence of any residual GO.

Raman spectroscopy provides a useful tool to determine the defect density and disorder in graphene related materials. The characteristic Raman spectrum of graphite shows a G peak (~1580 cm^−1^) that is associated with the *E*_2g_ stretching vibrational mode of sp^2^ C atoms plus an additional small D peak (~1350 cm^−1^) associated with defective sp^3^ C atoms [47]. This latter peak is absent in the non-defective graphene and only becomes active in the presence of disorder. From the intensities of these two bands, one can determine the I_D_/I_G_ ratio and use this parameter as a measure of the defect degree. However, this ratio does not always reflect the degree of reduction because defects can also be due to edges, ripples, or vacancies. The specific disorder or the degree of defects can be related to in-plane vacancies or impurities as interstitial or edge heteroatoms, or to a larger edge contribution consequence of a more reduced sheet size. The type of disorder or defects depends, to a large extent, on the reduction methodology employed [44].

**Figure 5:**
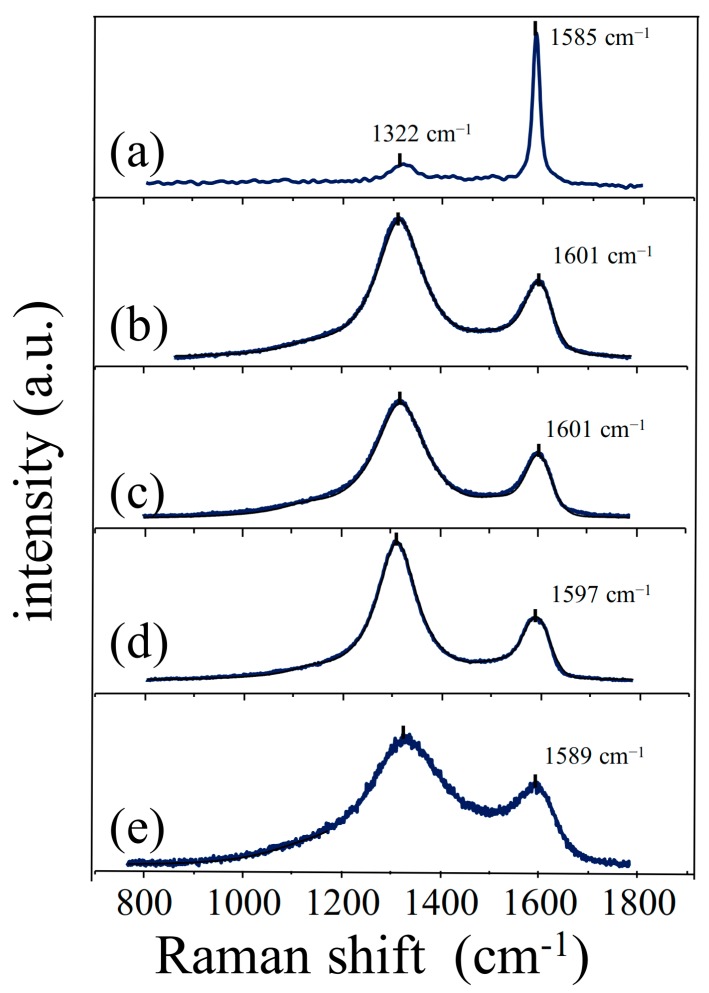
Raman spectra of (**a**) graphite; (**b**) CRGO; (**c**) hTRGO; (**d**) ERGO; and (**e**) TRGO.

Graphite (Figure 5a) shows a sharp and intense G peak at 1585 cm^−1^ and a very weak D peak at 1322 cm^−1^ caused by the graphite edges. In the RGO samples (Figure 5b–e), the D peak intensity increased with respect to that of the G peak in all four materials, indicating that the reduction of GO produced a significant degree of structural disorder. The evident peak broadening is also indicative of a large degree of disorder. As far as the positions of the peaks are concerned, a shift to higher frequencies (blue shift) of the G peak is observed for the CRGO, hTRGO, and TRGO, moving from the value of 1585 cm^−1^, which is characteristic of graphite, to those of 1601, 1601, and 1597 cm^−1^, respectively. Only for ERGO is the G peak located almost at the same frequency as it is in graphite (1589 cm^−1^). Considering that all measurements were performed using the same laser excitation frequency (785 nm), shifts due to changes in the laser frequency are excluded. According to Kudin et al. [48], the blue shift is mainly due to the presence of isolated double bonds that resonate at higher frequencies than the G peak of graphite. In ERGO, the G peak appears at the same position as the G peak in graphite; Kudin attributes this to a graphitic “self-healing”, which restores most of the in-plane sp^2^-bonding network [48]. This implies that this “self-healing” occurs in a more pronounced manner during the electrochemical reduction process than during the other reductive methods [18]. The I_D_/I_G_ ratios were calculated from the intensity of the peaks observed in the spectra of the different RGO materials. CRGO and hTRGO show similar values (1.82 and 1.80, respectively), whereas ERGO shows the highest value (2.20) and TRGO shows the lowest value (1.57). All of the I_D_/I_G_ ratios obtained in this work are larger than those reported by Sakinyte et al., Mohan et al., and Mei et al., whose values range between 1.0 and 1.2 for thermal, hydrothermal, and chemical RGOs, respectively [18,45,46]. The larger values obtained in the present work can be associated with a higher degree of disorder, which correlates well with the loss of crystallinity observed by XRD. Additionally, the broad peaks observed in the Raman spectrum of TRGO suggest that TRGO presents a remarkable amount of order with respect to the total crystallite area. This is in good agreement with both the TEM and BET results that revealed a sheet size lower than 1 µm and at a large surface area [36], as well as with SECM and voltammetric characterization.

### 3.4. Electrochemical Behaviour of an Oligonucleotide on RGOs Modified Electrodes

**Figure 6:**
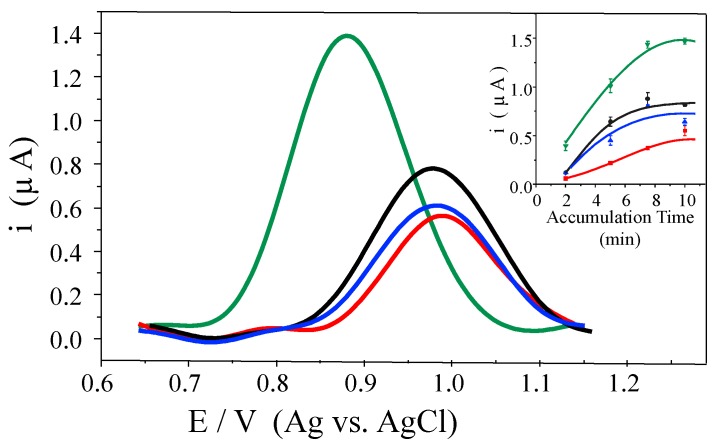
Differential pulse voltammograms obtained in 0.1 M PBS (pH 7.0) using a GCE modified with CRGO (red); hTRGO (black); ERGO (blue); and TRGO (green) (1.0 mg/mL) after 10 min accumulation of the Oligo solution. Inset: Dependence of the guanine oxidation current with the accumulation time from a 15 µM Oligo solution at an open circuit potential.

The DNA sensing was monitored by differential pulse voltammetry (DPV) through the current oxidation of a guanine base, which has the lowest oxidation potential of all DNA bases [49]. Figure 6 shows the DP voltammograms obtained for 15 µM Oligo after 10 min of accumulation time at the GCE/CRGO, GCE/hTRGO, GCE/ERGO, and GCE/TRGO electrodes. The voltammograms are presented in the baseline-subtracted form for a better comparison. It can be observed that all of the RGOs produce a well-resolved and intense peak, but clearly, the highest signal was obtained by using the GCE/TRGO electrode. The currents for the graphene-based electrodes were 1.39, 0.78, 0.61, and 0.56 µA for GCE/TRGO, GCE/hTRGO, GCE/ERGO, and GCE/CRGO, respectively. In addition, a significant shift to a lower potential value was observed when GCE/TRGO was used (0.881 V) compared to the shifts obtained for GCE/hTRGO, GCE/ERGO, and GCE/CRGO (0.980, 0.984, and 0.989 V, respectively). The best current response using TRGO was observed independently of the accumulation times (inset Figure 6), and the same behaviour was observed for the oxidation potential (not shown). In a previous study, we compared the effect of using carbon nanotubes or graphene, either oxidized or reduced, on the electrochemical behaviour of double strand DNA (dsDNA), and no potential shift was observed when oxidized and non-oxidized multi-walled nanotubes (MWNT, MWNT-OX), and a commercially available chemically reduced GO were used; furthermore, when GO was used, a 40 mV more positive potential shift was observed [29].

Finally, at the maximum response obtained for each RGO (inset Figure 6), accumulation times of 7.5 min for hTRGO, ERGO, and TRGO and 10 min for CRGO were selected to perform the Oligo concentration study. The linear concentration ranges obtained were 2.5–15 µM for CRGO and hTRGO and 2.5–20 µM for ERGO and TRGO. As far as the sensitivity was concerned, values of 30, 40, 40, and 80 µA/mM for ERGO, hTRGO, CRGO, and TRGO, respectively, were obtained.

The results indicate that TRGO produces a fast electron transfer process evidenced in the 0.100 V potential shift towards low potential values, an almost two-fold increase in current intensity, and a higher sensitivity compared with the other three RGOs. Given the similarity of the XPS C 1s spectra of TRGO, hTRGO, and EQRGO, the improved behaviour of TRGO must not be related to the presence of a particular carbon functional group; that is, the explanation is not strictly or solely compositional. According to the Raman spectroscopy results, TRGO is not the material with the highest sp^2^ character of all the reduced materials. Therefore, the explanation should include a combination of compositional, structural, and morphological factors. Its large graphitic surface, its lower sheet size, and its high polydispersity, resulting in a high physical and electrochemical area with a great quantity of defects likely exposed at the borders, must be of crucial importance to explain the observed behaviour. Our results are in accordance with the results published by Dolbin et al. [17], who demonstrated that temperatures as high as those used in our study (900 °C) generate new defects on the TRGO surface.

## 4. Conclusions

We have synthesized and fully characterised four reduced graphene oxides produced by chemical, hydrothermal, electrochemical, and thermal methods. The reduction method produces materials with completely different characteristics such as sheet size, amount of remaining oxygen, defects, and electroactivity. Thus, the electron transfer shows how the electrochemical oxidation of DNA is strongly dependent on the reduction method used to created the reduced graphene oxide. The advantages of modifying GCE with TRGO instead of CRGO, hTRGO, and ERGO are clearly demonstrated. Thus, the thermal method produces a reduced graphene oxide material with the best features to electrochemically oxidize DNA due to its high surface area with a great quantity of defects likely exposed on the borders, which induces the highest electroactivity among the evaluated RGOs.

## Supplementary Materials

The following are available online at http://www.mdpi.com/2079-4991/7/7/168/s1, Figure S1: wide scan XPS spectra, Figure S2: TEM image, Figure S3: XRD patterns of graphite, graphene oxide and chemical hydrothermal, electrochemical and thermal RGO.
