# Supplementary material for: Reduced Graphene Oxides: Influence of the Reduction Method on the Electrocatalytic Effect towards Nucleic Acid Oxidation
Source: Nanomaterials (Basel). 2017 Jul 4;7(7):168. doi: 10.3390/nano7070168 (PMC5535234; doi:10.3390/nano7070168)
Supplement: Supplementary file 1 [file nanomaterials-07-00168-s001.pdf]

Supporting Information for

Reduced Graphene Oxides: Influence of the Reduction Method on the Electrocatalytic Effect towards Nucleic Acid Oxidation

Daniela F. Báez [1,2], Helena Pardo [3], Ignacio Laborda [3], José F. Marco [4], Claudia Yáñez [1] and Soledad Bollo [1,2,*]

[1] Centro de Investigación de Procesos Redox, CiPRex,Facultad de Ciencias Químicas y Farmacéuticas, Universidad de Chile, Sergio Livingstone 1007. Independencia, Santiago 8380492, Chile; d.baez@ciq.uchile.cl (D.F.B.); cyanez@ciq.uchile.cl (C.Y.)

[2] Advanced Center for Chronic D (ACCDiS), Facultad de Ciencias Químicas y Farmacéuticas, Universidad de Chile, Sergio Livingstone 1007. Independencia, Santiago 8380492, Chile

[3] Facultad de Química, Universidad de la República de Uruguay, Avenida General Flores 2124, Montevideo 11800, Uruguay; hpardo@fq.edu.uy (H.P.); ilaborda@fq.edu.uy (I.L.)

[4] Instituto de Química Física Rocasolano, CSIC, Madrid 28006, Spain; jfmarco@iqfr.csic.es (J.F.M)

* Correspondence: sbollo@ciq.uchile.cl

Keywords: graphene; reduced graphene oxide; glassy carbon electrode; SECM; DNA oxidation

Conflicts of Interest: The authors declare no conflict of interest.

Electronic Supplementary Information. Báez et al

Figure 1S: X-ray photoelectron spectroscopy (XPS). Wide scan XPS spectra recorded from (**a**) Graphite; (**b**) GO; (**c**) CRGO; (**d**) hTRGO; (**e**) ERGO and (**f**) TRGO.

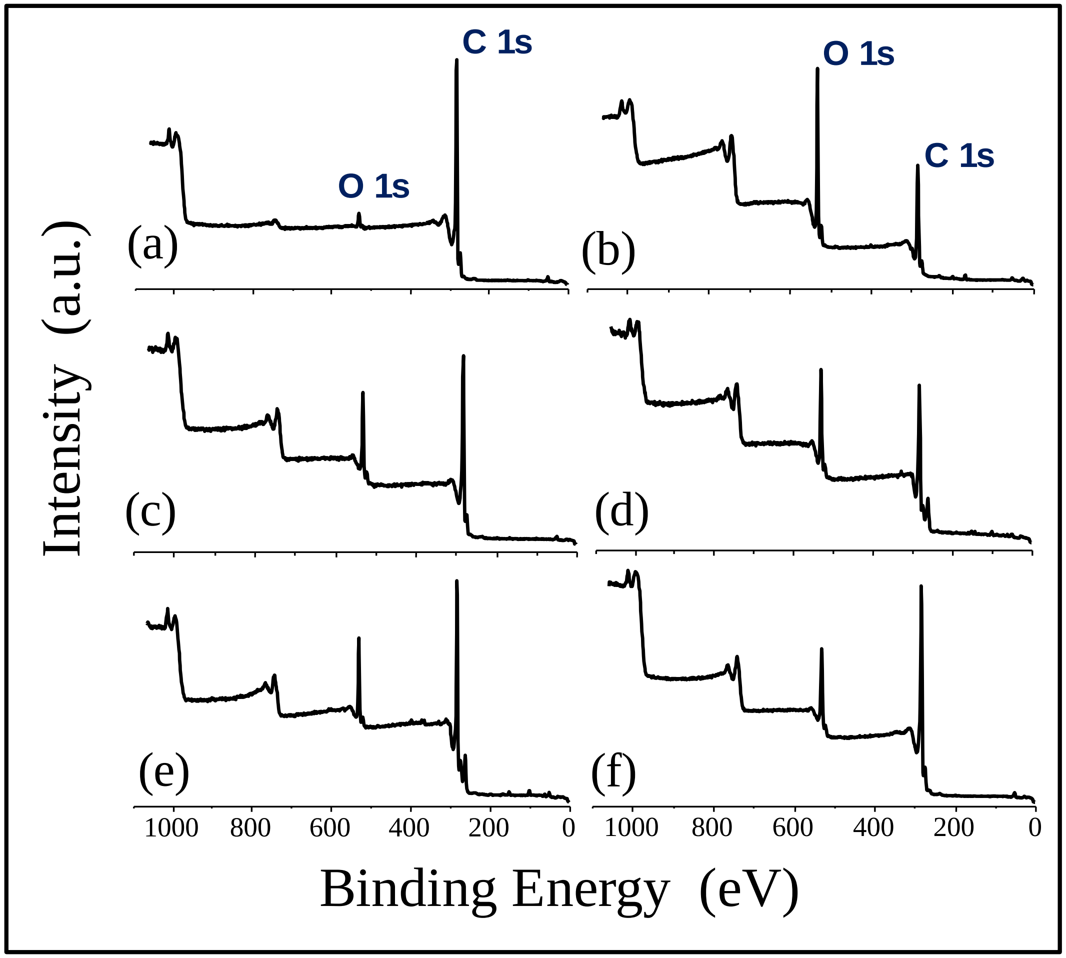

Figure 2S: Transmission electron microscopy, TEM images of (**a**) GO; (**b**) CRGO; (**c**) hTRGO; (**d**) ERGO and (**e**) TRGO.

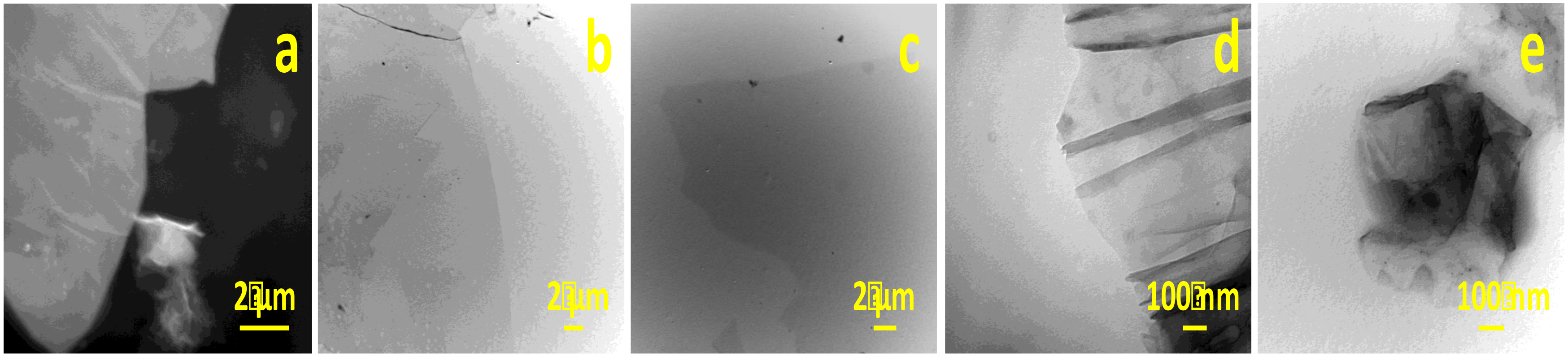

TEM images were obtained with a JEOL JEM-1010 microscope, operating at 100 kV. The RGOs were dispersed in ethanol, sonicated for 30 minutes and deposited on a carbon/copper 200-mesh grid.

Figure 3S: X-ray Diffraction, XRD patterns of (**a**) Graphite; (**b**) GO; (**c**) CRGO; (**d**) hTRGO; (**e**) ERGO and (**f**) TRGO.

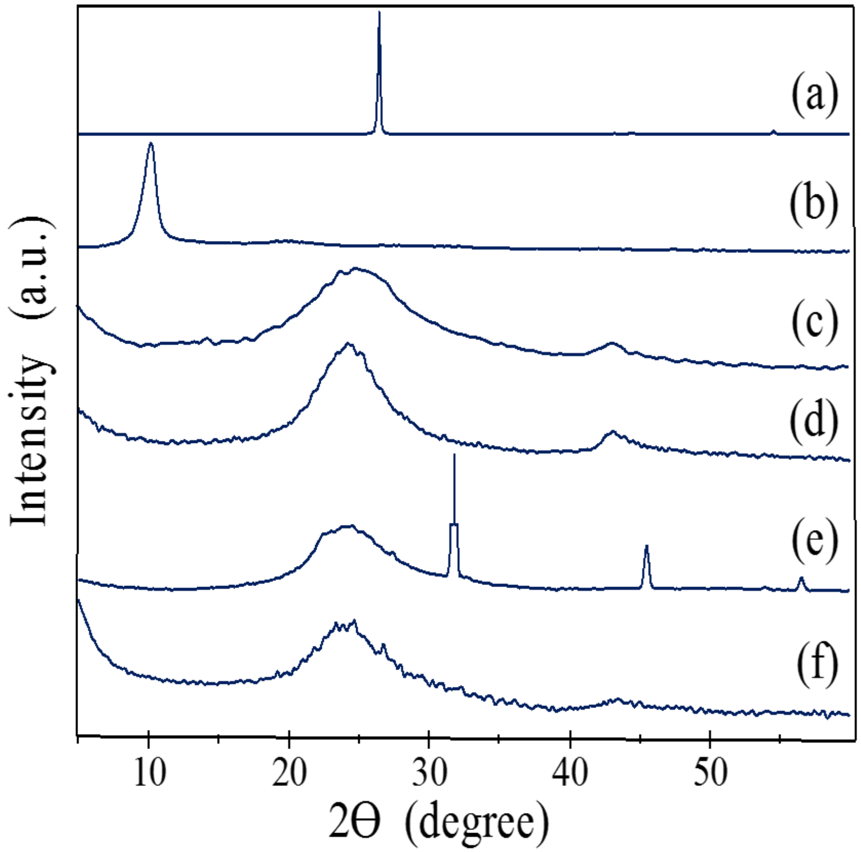

XRD diffractograms were performed using a Rigaku Ultima IV diffractometer using Cu$K\alpha$ radiation (λ = 1.5418 Å) in the 2θ = 5- 60° range
